# Supplementary material for: Nonadditive gene expression is correlated with nonadditive phenotypic expression in interspecific triploid hybrids of willow (Salix spp.)
Source: G3 (Bethesda). 2021 Dec 21;12(3):jkab436. doi: 10.1093/g3journal/jkab436 (PMC9210313; doi:10.1093/g3journal/jkab436)
Supplement: jkab436_Supplemental_Figures [file jkab436_supplemental_figures.pdf]

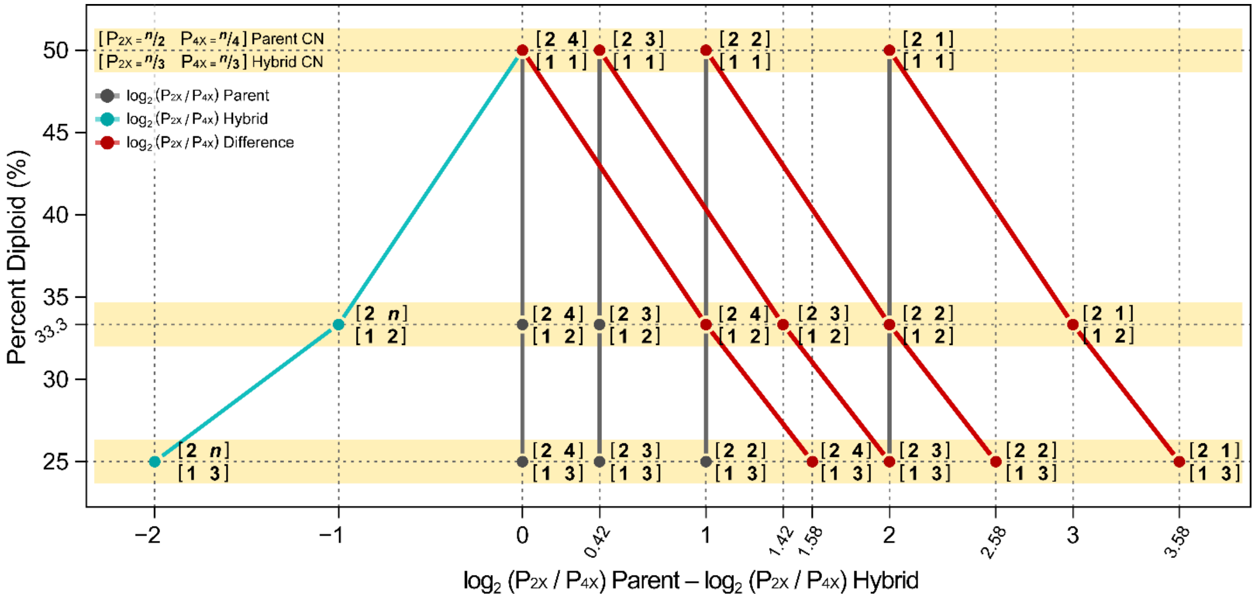

Figure S1. Model schematic comparing parent allele-specific $\log_2(P_{2X} / P_{4X})$ with the percentage of reads in a triploid hybrid attributable to the diploid parent (i.e., diploid %). Lines represent the $\log_2(P_{2X} / P_{4X})$ in the hybrid (cyan), parent (dark grey), and the parent–hybrid difference (red). Brackets to the right of each point represents the copy number (CN) out of the total expected to occur in a diploid, triploid, or tetraploid state, e.g. [**2** **4**] = [P_{2X} = **2**/2 *and* P_{4X} = **4**/4] in the parents and [**1** **2**] = [P_{2X} = **1**/3 *and* P_{4X} = **2**/3] in the hybrid.

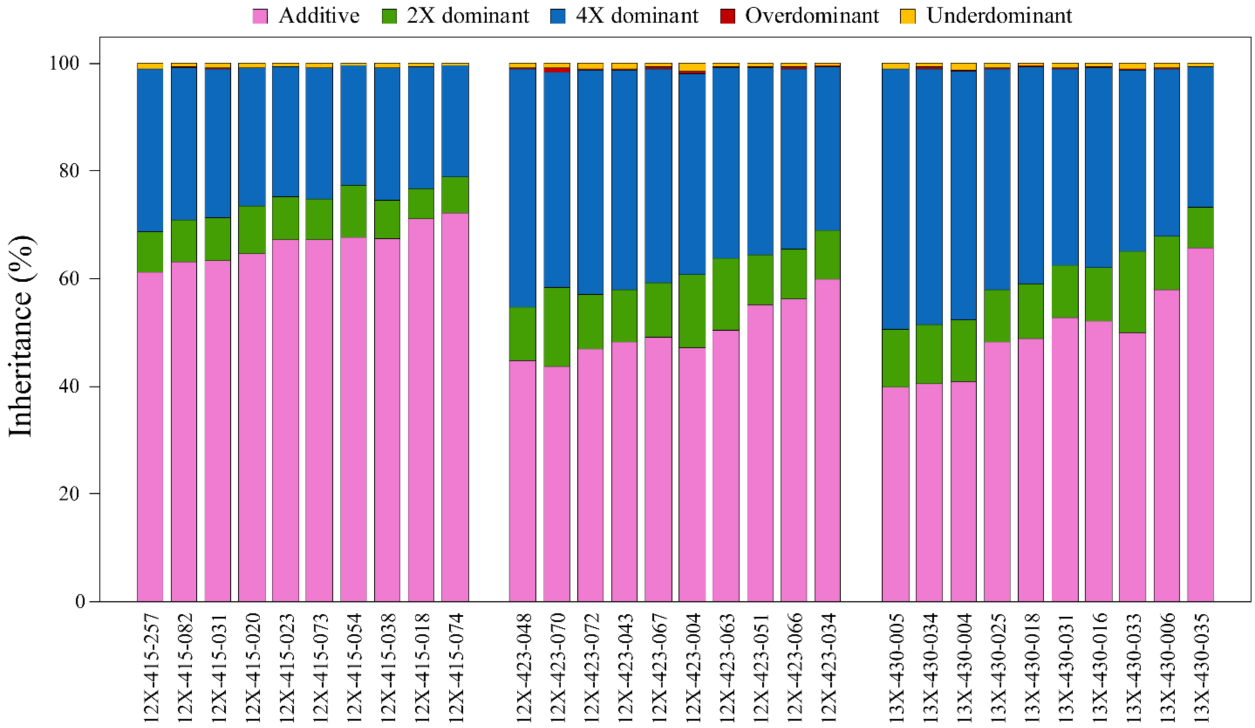

Figure S2. Percentage of additive and nonadditive genes in triploid individuals. Only genes differentially expressed between the parents (not conserved or ambiguous) were used in to calculate inheritance class percentages. Gene expression inheritance patterns are colored according to the legend to the right of the graph. Individuals are shown in order of their additive (%) of differentially expressed genes.

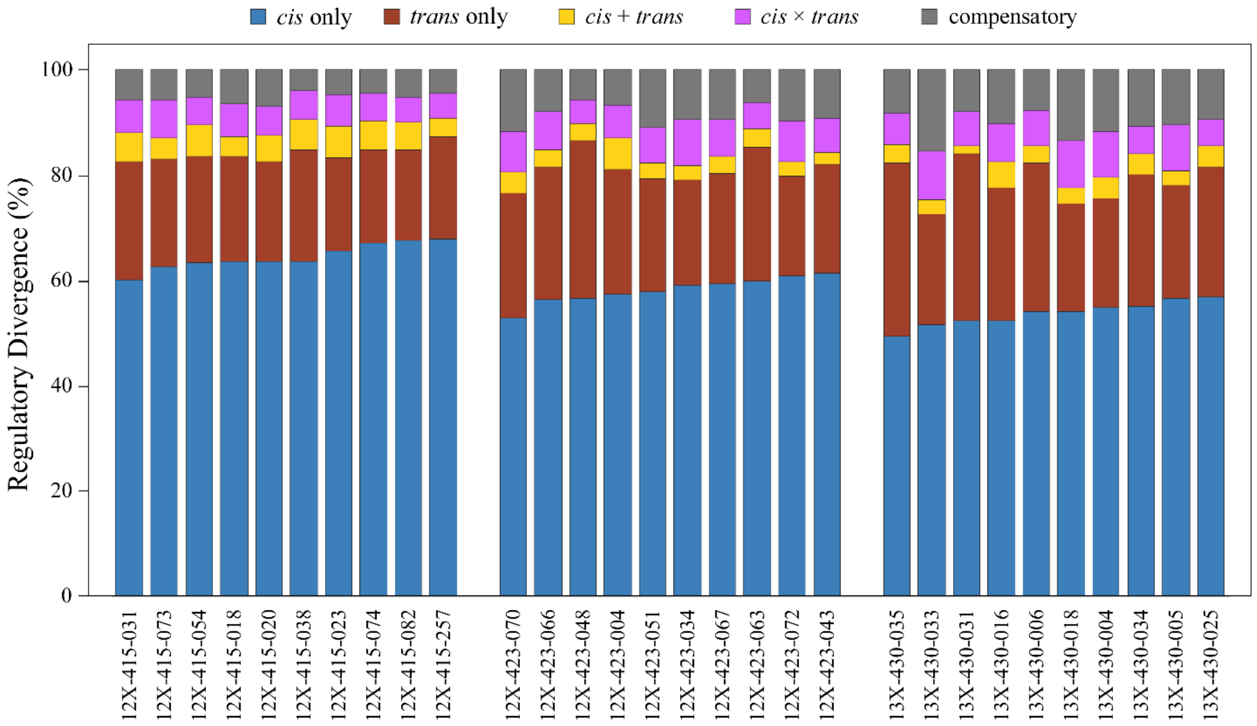

Figure S3. Regulatory divergence percentages by class. Only genes showing allele-specific expression (not conserved or ambiguous) are depicted. Classes are colored according to the legend above the graph. Individuals within families are ordered according to cis (%).

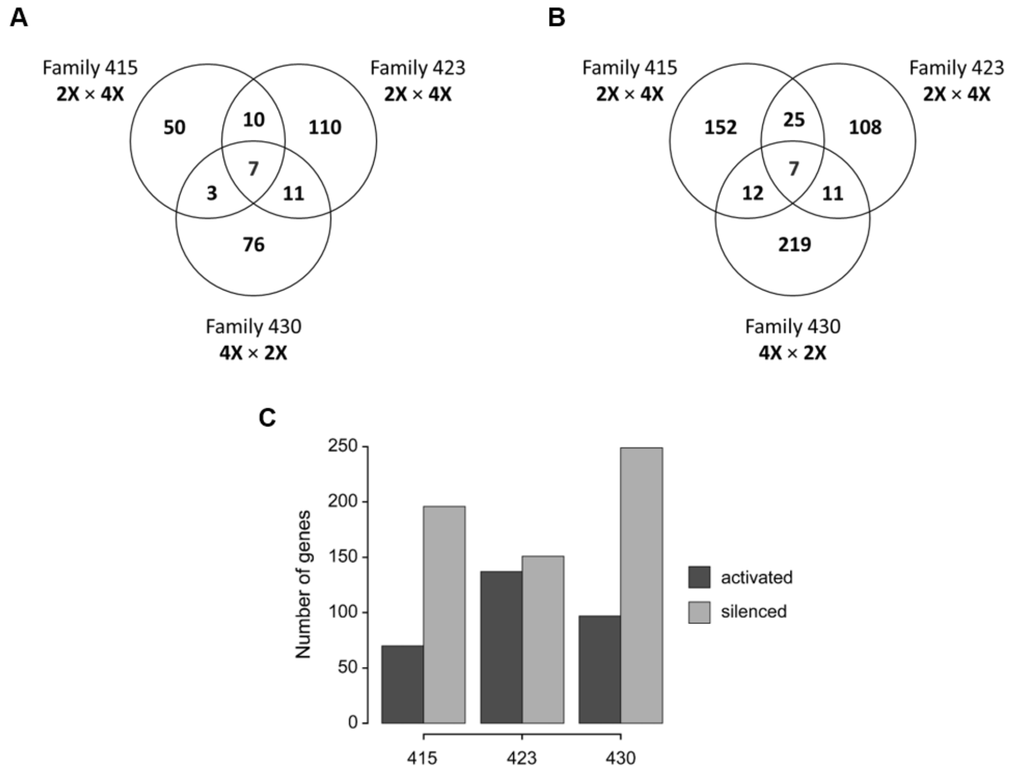

Figure S4. Number of genes activated and silenced in triploids and parents. The Venn diagram in **(A)** illustrates genes activated in hybrids and silenced in the parents, whereas the Venn diagram in **(B)** illustrates genes activated in the parents and silenced in the hybrids. The barplot **(C)** summarizes the total number of genes activated (dark grey bars) or silenced (light grey bars) for each family.
